# The Digital Divide Among Low-Income Homebound Older Adults: Internet Use Patterns, eHealth Literacy, and Attitudes Toward Computer/Internet Use

**DOI:** 10.2196/jmir.2645

**Published:** 2013-05-02

**Authors:** Namkee G Choi, Diana M DiNitto

**Affiliations:** ^1^The University of Texas at AustinAustin, TXUnited States

**Keywords:** homebound older adults, housebound older adults, Internet, eHealth literacy, attitudes toward Internet

## Abstract

**Background:**

Internet technology can provide a diverse array of online resources for low-income disabled and homebound older adults to manage their health and mental health problems and maintain social connections. Despite many previous studies of older adults’ Internet use, none focused on these most vulnerable older adults.

**Objective:**

This study examined Internet use patterns, reasons for discontinued use, eHealth literacy, and attitudes toward computer/Internet use among low-income homebound individuals aged 60 and older in comparison to their younger counterparts—homebound adults under age 60.

**Methods:**

Face-to-face or telephone surveys were conducted with 980 recipients of home-delivered meals in central Texas (78% were age 60 years and older and 22% under age 60). The eHealth Literacy Scale (eHEALS) and the efficacy and interest subscales of the Attitudes Toward Computer/Internet Questionnaire (ATC/IQ) were used to measure the respective constructs. Age groups were compared with chi-square tests and *t* tests. Correlates of Internet use were analyzed with multinomial logistic regression, and correlates of eHEALS and ATC/IQ scores were analyzed with OLS regression models.

**Results:**

Only 34% of the under-60 group and 17% of the 60 years and older group currently used the Internet, and 35% and 16% of the respective group members reported discontinuing Internet use due to cost and disability. In addition to being older, never users were more likely to be black (OR 4.41; 95% CI 2.82-6.91, *P*<.001) or Hispanic (OR 4.69; 95% CI 2.61-8.44, *P*<.001), and to have lower incomes (OR 0.36; 95% CI 0.27-0.49, *P*<.001). Discontinued users were also more likely to be black or Hispanic and to have lower incomes. Among both age groups, approximately three-fourths of the current users used the Internet every day or every few days, and their eHEALS scores were negatively associated with age and positively associated with frequency of use. Among the 60 and older group, a depression diagnosis was also negatively associated with eHEALS scores. ATC/IQ efficacy among never users of all ages and among older adults was positively associated with living alone, income, and the number of medical conditions and inversely associated with age, Hispanic ethnicity, and Spanish as the primary language. Although ATC/IQ interest among older adults was also inversely associated with age, it was not associated with Hispanic ethnicity and Spanish as the primary language.

**Conclusions:**

This study is the first to describe in detail low-income disabled and homebound adults’ and older adults’ Internet use. It shows very low rates of Internet use compared to the US population, either due to lack of exposure to computer/Internet technology; lack of financial resources to obtain computers and technology; or medical conditions, disabilities, and associated pain that restrict use. Recommendations to reduce the digital divide among these individuals are provided.

## Introduction

With the unprecedented growth of the aging population, the number of disabled and homebound older adults continues to increase. Census data for 2009 show that 23.5% of the 40 million noninstitutionalized adults aged 65 years and older in the United States had an ambulatory disability and 15.8% had an independent living disability [1]. The Centers for Medicare and Medicaid Services also report that in 2011, 3.3 million Medicare or Medicaid recipients aged 65 and older received home health care services [2]. Of disabled and homebound older adults, the oldest, poorest, and physically and mentally most vulnerable tend to receive home-delivered meals (HDM, commonly known as “Meals on Wheels”) funded under Title III of the Older Americans Act [3]. In 2009, a little over 880,000 older adults (9%, 60-64 years old; 22%, 65-70 years old; 40%, 75-84 years old; and 30%, 85 years old and up) received HDM [4,5]. These low-income homebound older adults, who often have multiple chronic medical conditions and disabilities, face daunting tasks maintaining their independent living status. Their mobility impairments and lack of financial resources are significant barriers to conducting basic activities of daily living and instrumental activities of daily living (ADLs/IADLs) and remaining socially engaged, and as a result, they also become highly vulnerable to depression [6-8].

Internet technology can provide a diverse array of online resources for homebound older adults to manage their health and mental health problems and ADLs/IADLs. For example, they can use the Internet to search for health information; participate in freely available online chronic disease self-management programs, health and mental health support groups, and exercise programs; and order medications, make appointments, and communicate with their health care providers. Homebound older adults may also be able to stay connected to their close social support networks and the larger community via emails and video calls and by visiting religious sites, social networking sites, chat/discussion groups, interest/hobby groups, and news and blog sites. They may also take advantage of online shopping, banking, and bill paying to take more control of their daily lives. Furthermore, Internet access can allow them to benefit from the growing number of telehealth and telemental health interventions [9-11].

Older-adult Internet users indeed report multiple benefits of using this technology including convenience of accessing health-and nonhealth-related information; increased communication and social connections with family, friends, and others regardless of geographical distance; keeping abreast of news and other happenings in their immediate and global communities; participating in a variety of online educational, social, and recreational activities; and convenience of online shopping, banking, and travel arrangements [12-18]. Internet technology and web-based resources may likewise promote homebound older adults’ physical and mental health and reduce their social isolation and dependence on informal and formal support systems.

Although older adults are the fastest growing group of Internet users, their use still lags behind all other age groups. In April 2012, the Pew Internet and American Life Project found that among a nationally representative sample of Americans, 53% of those aged 65 and older, but only 34% of those aged 76 and older, used the Internet or email, in contrast to 97% of the 18-29 age group, 91% of the 30-49 age group, and 77% of the 50-64 age group [19]. Given their advanced age, high degree of functional impairment, limited financial resources, and social isolation, Internet use among low-income homebound older adults is likely to be even lower than that among general older adults. In fact, previous studies found that older adults who did not use the Internet or email tended to be older and of racial/ethnic minority status and had less education, worse physical and functional health, fewer social and financial resources, and greater loneliness/perceived social isolation [20-25]. Other studies also report that the most powerful predictors of not using information technology among older adults are cognitive decline associated with aging processes and attitudes such as anxiety about computer use and the perception that the technology was not useful for them [14,25,26]. Previous studies have not found consistent evidence about depressive symptoms as a correlate of older adults’ computer/Internet use. One study found depressive symptoms to be negatively associated with Internet use [27]; another found no relationship [25].

Older adults themselves report the following reasons for not using computer/Internet technology: the cost of the computer/other equipment and Internet access, functional impairments such as arthritis and joint pain that interfere with typing, visual deficits, ergonomic barriers (eg, small font sizes), lack of computer knowledge, lack of computer-efficacy (beliefs about their ability to use computers/Internet technology) and general self-efficacy (eg, “too old to learn new things”), and mistrust of Internet systems and privacy-related concerns [15,21,28]. However, previous studies also found that experience with computers/Internet reduced anxiety and increased self-confidence and positive attitudes about computers/Internet use in older adults regardless of income and educational levels [18,29-32].

For older adults in general and for low-income, disabled, homebound older adults in particular, eHealth literacy, along with Internet access, is an important dimension of the digital divide, ie, those who use versus those who do not use this technology. eHealth literacy is “the ability to seek, find, understand and appraise health information from electronic sources and apply knowledge gained to addressing or solving a health problem” and is composed of basic literacy, health literacy, scientific literacy, media literacy, and computer literacy [33]. Previous studies show that (1) eHealth literacy is lower among older adults, those with lower socioeconomic status, and those with less computer experience, and (2) higher eHealth literacy is associated with more positive outcomes from Internet searches in three domains: cognitive (eg, health knowledge/information gathering), instrumental (eg, self-management of health needs and health behaviors), and interpersonal (eg, interactions with physicians) [34-36]. Studies also find that health information technology training among older adults results in significant gains in eHealth literacy and ability to navigate complex health websites [18,34]. Government agencies and many private nonprofit and for-profit sector organizations now make prodigious amounts of health and mental health information and resources available online. Given vulnerable low-income homebound older adults’ substantial health and mental needs, examining their ability to search for high-quality health information/resources and make informed decisions about applying the information to improve their quality of life may be particularly useful. This examination could identify their training needs to help close the digital divide and allow them to reap the multiple benefits of Internet and computer technology use.

Given that no study has examined Internet use patterns, eHealth literacy, and attitudes toward computer/Internet among low-income homebound older adults (aged 60 and older), we did so by comparing them with their younger counterparts—low-income homebound adults under age 60. The research questions in the present study were: Compared to their younger counterparts, (1) What are the rates of current Internet use, previous use, and never use among older adults?, (2) Among current users, what is the frequency of Internet use, types of Internet activities in which they engage, physical and functional difficulties using the Internet, and their comfort level in joining online health discussion groups and exchanging emails with other participants?, (3) Among previous users, what are the reasons for discontinued use?, and (4) Among never users, what is their level of willingness and comfort to engage in conducting health-information searches and joining online health discussion groups and exchanging emails with other participants? In addition, we tested the following hypotheses: Controlling for age, (H1) Never users and previous users will be more likely than current users to be black or Hispanic, lack English proficiency, have lower income, have more chronic medical conditions and ADL/IADL impairments, and have self-reported diagnoses of depression and anxiety, (H2) Among current users, eHealth literacy will be lower among those with self-reported diagnoses of depression and anxiety and lower frequency of Internet use, and (H3) Among never users, attitudes toward computer/Internet use (efficacy and interest) will be positively associated with higher income and living alone and negatively associated with lack of English proficiency and self-reported diagnoses of depression and anxiety.

## Methods

### Participants and Procedures

The data are from a survey of 980 HDM recipients residing in central Texas. The HDM program is operated by a multipurpose aging-service agency that serves about 2100 low-income, disabled, and homebound adults (80% were 60 years and older, and 20% under age 60) daily. The agency receives partial funding for HDM and case management services for clients aged 60 and older under Title III of the Older American Act and partial funding for the same services for persons under age 60 from the state Medicaid program and the Social Services Block Grant. The computer/Internet use survey was conceived as part of the agency’s strategic exploration of the potential for using emails as part of its communication with clients and for providing case management services via videoconferencing in the future. Through an academic and community collaborative partnership, one of the authors helped the agency develop the survey questionnaire and trained the HDM program’s 12 full-time case managers to conduct the survey with their clients. Most surveys were done between November 2012 and February 2013, either in person using the paper form survey questionnaire or by telephone using the electronic form survey questionnaire. The Spanish version of the survey was used for the clients who spoke Spanish only (< 3% of respondents). Clients assessed as having moderate to severe cognitive impairment based on the 4-item (memory, concentration, orientation, and decision-making) cognition test contained in the HDM program’s intake and recertification assessments were excluded from the survey. Clients unable to participate in the survey due to severe mental illness and those who refused to participate for any reason were also excluded. All survey data were entered in the agency’s centralized electronic client data management system and linked with each respondent’s intake or most recent recertification assessment data. With approval from the University of Texas at Austin’s Institutional Review Board, the de-identified data were analyzed in March 2013.

### Measures

#### Internet Use

Internet use was measured with the question, “Have you ever used the Internet?” The answer categories were (1) No, I have never used it (never user), (2) I have used it before but not currently (previous user), and (3) Yes, I am a current user. The previous users (n=75 adults under age 60 and n=120 adults aged 60 or older) were asked the reasons for discontinuation (no computer or Internet connection at home because of cost, it is not helpful, I do not need it, cannot use computer because of disability or pain, and other—specify).

#### Internet Use Patterns and Activities

Patterns and activities in Internet use among current users (n=73 adults under age 60 and n=128 adults aged 60 or older) were ascertained with the following items: (1) location of Internet connection (at home, apartment complex, family/friend’s home, and other—specify) and frequency of Internet use (at least once a day, every few days, once a week, a few times a month, once a month or less often), (2) type of activities conducted on the Internet (research health-related information, research information about other topics or issues of interest, send/receive email, buy products online, do banking online and/or pay bills, read news, papers, magazines, and books online, play games online, watch videos (including YouTube), use social networking or dating site (eg, Facebook, Match.com), and other—specify, (3) ease/difficulty of locating websites the user was looking for and finding the information that he/she needed within that site (on a 5-point Likert scale: 1=“always easy” to 5=“very difficult”), and (4) any physical/functional problem that made it harder for the respondent to use the Internet (pain in the limbs, unsteady hands, difficulty concentrating for long periods of time, difficulty sitting for long periods, eyes that tire easily, and other—specify).

#### eHealth Literacy

eHealth Literacy among current Internet users was measured by the 8-item eHealth Literacy Scale (eHEALS) with each item scored on a 5-point Likert scale. The eHEALS measures the concept of eHealth literacy as defined as a set of skills required to effectively engage information technology for health and has shown high levels of internal consistency and good test-retest reliability [33]. The items are: (1) I know *what* health resources are available on the Internet, (2) I know *where* to find helpful health resources on the Internet, (3) I know *how* to find helpful health resources on the Internet, (4) I know *how to use* the Internet to answer my questions about health, (5) I know how to use *the health information* I find on the Internet to help me, (6) I have the skills I need to *evaluate* the health resources I find on the Internet, (7) I can tell *high-quality* health resources from *low-quality* health resources on the Internet, and (8) I feel *confident* in using information from the Internet to make health decisions. The final eHEALS score is the average of all 8 items, with higher scores suggesting higher eHealth literacy. The internal consistency reliability in the original eHEALS validation study with a sample of 664 individuals aged 13-21 was Cronbach alpha=.88, and the principal components analysis produced a single factor solution (factor loadings ranging from .60 to .84 among 8 items; eigenvalue=4.479; and 56% of the variance explained). Item-scale correlations ranged from *r*=.51 to *r*=.76, and test-retest reliability showed modest stability over a 6-month period (*r*=.49 to *r*=.68) [33]. Although the eHEALS was originally validated with adolescents and young adults, it has been used to measure eHealth literacy among older adults [37]. In the present study, internal consistency reliability coefficient for the 8-item eHEALS for current Internet users was Cronbach alpha=.93 for both the younger (under age 60) group (n=73) and the older (60 years and up) group (n=128) adults.

In addition to these 8 items, two eHEALS supplemental items were used to measure (1) perceived usefulness of the Internet in helping make health decisions, and (2) perceived importance of being able to access health resources on the Internet. Both items were scored on a 5-point Likert scale with higher scores suggesting higher levels of perception. eHEALS developers recommend using these two supplemental items along with the 8 items.

#### Attitudes Toward Computer/Internet

Among never users (n=69 adults under age 60 and n=515 adults aged 60 or older), attitudes toward computers/Internet were measured with the 5-item computer efficacy subscale and the 5-item computer interest subscale of the Attitudes Toward Computers Questionnaire (ATCQ), with each item scored on a 1-5 point Likert scale. The ATCQ was originally validated to measure seven dimensions of attitudes toward computers (comfort, efficacy, gender equality, control, dehumanization, interest, and utility) among 398 students in Grades 4 through 12 [38]. It was later validated with 420 older adults and used to assess older adults’ attitudes toward computers in previous studies [30,37,39,40]. In the present study, the original wording “computer” was changed to “computer/Internet” in each item (ATC/IQ hereafter) to emphasize the Internet. The efficacy items were (1) I know that if I worked hard to learn about computers/Internet, I could do well, (2) Computers/Internet are *not* too complicated for me to understand (*italics* in original), (3) I think I am the kind of person who would learn to use a computer/Internet well, (4) I think I am capable of learning to use a computer/Internet, and (5) Given a little time or training, I know I could learn to use a computer/Internet. The interest items were (1) Learning about computers/Internet is a worthwhile and necessary subject, (2) Reading or hearing about computers/Internet would be (is) boring, (3) I don’t care to know more about computers/Internet, (4) Computers/Internet would be (are) fun to use, and (5) Learning about computers/Internet is a waste of time. For both efficacy and interest subscales, the final score is the average of all 5 items, and higher scores suggest higher computer/Internet efficacy or interest. In the present study, the internal consistency reliability coefficients for the efficacy subscale were Cronbach alpha=.85 for those under age 60 and .92 for those age 60 and up. The internal consistency reliability coefficients for the interest subscale were .88 for those under age 60 and .84 for those age 60 and up.

#### Willingness to Use Online Health Information

Among never users, willingness to use online health information was measured with one item, “If someone can teach me how to use the Internet to look for health information, I am willing to try” and scored on a 1-5 point Likert scale. Higher scores suggest greater willingness.

#### Comfort With Joining Online Health Discussion Groups and Exchanging Emails With Other Participants

*A*mong both current and never users, comfort with joining online health discussions and exchanging emails with other participants was measured with one item, “I would be comfortable joining an online health discussion group and exchanging emails with other participants” and scored on a 1-5 point Likert scale. Higher scores suggest greater levels of comfort.

#### Sociodemographics

Sociodemographics included age in years, gender, race/ethnicity (nonHispanic white—reference category, black/African American, Hispanic, and other), marital status (married, widowed, divorced/separated, and never married), living arrangement (living alone, living with spouse [and any other person], living with another adult, and living with dependent adult child[ren] or minor child[ren] only), income-to-needs ratio (ratio of income to the official poverty line adjusted for the number of family members, with higher ratios indicating higher income/better financial situation), and primary language the respondent speaks at home (English, both English/Spanish, Spanish only). The agency assessment does not include any questions about the client’s level of education.

#### Health, Mental Health, and Disability

Health, mental health, and disability were measured by (1) the number of chronic medical conditions (arthritis, hypertension, diabetes, heart disease, lung disease, kidney disease, stroke, and cancer) that the respondent reported as having been diagnosed by a doctor, (2) diagnoses of depression, anxiety, and severe mental illnesses that the respondent reported (and confirmed by case managers using the respondent’s list of medications when possible), and (3) the number of ADL impairments (feeding/eating, bathing, grooming, dressing, toileting, and getting in and out of bed) and IADL impairments (cleaning, preparing meals, doing laundry, grocery shopping, making telephone calls, and taking medications).

### Analysis

First, we examined data integrity and missingness using univariate frequency distributions. Of 983 surveys conducted, 3 had incomplete data, leaving 980 usable surveys. Then we examined survey participants’ representativeness by comparing their sociodemographic characteristics to those of all HDM clients the agency serves. Despite the exclusion of cognitively impaired and some severely mentally ill clients, survey participants’ sociodemographic characteristics did not differ statistically from all HDM clients. Next, we conducted bivariate analyses using chi-square and *t* tests to compare respondents under 60 years of age with respondents 6 years of age and over in terms of their computer/Internet use patterns, reasons for discontinued use, eHealth literacy, attitudes toward computer/Internet use, and their willingness and comfort level regarding joining online health discussion groups and exchanging emails with other participants. Finally, we used multinomial logistic regression models to test H1 (Internet use/previous use/never use) and ordinary least squares (OLS) regression models to test H2 (correlates of eHealth literacy) and H3 (computer/Internet efficacy and interest), with sociodemographic and health/mental health characteristics as covariates. To examine correlates that may be specific to older adults (age 60 and up), we also ran separate multivariate models for them. In the multivariate regression analyses, 12 respondents who were not nonHispanic white, black, or Hispanic were excluded because of their small number. All statistical analyses were conducted with SPSS v.20.

## Results

### Participant Characteristics

Table 1 shows that the participants consisted of 217 (22.1%) people under age 60 and 763 (77.9%) people aged 60 years or older, 70% were female and 30% male, and 42% were nonHispanic white, 36% black, and 21% Hispanic. (The younger group ranged in age from 30-59 years, with 75% at 50-59. The oldest person in the older age group was 102 years old.) Participants’ median income-to-needs ratio was 1.15, they had an average of three chronic illnesses, 42% reported a depression diagnosis, and 23% reported an anxiety diagnosis. Participants were indeed low-income and had high levels of physical, functional, and mental distress. The two age groups did not differ in terms of gender, racial/ethnic distributions, and number of ADL impairments, while the older age group had greater income and higher numbers of chronic illnesses and IADL impairments but lower rates of self-reported diagnoses of depression, anxiety, and severe mental illness. The majority of both groups lived alone, but their living arrangements and marital status differed (eg, the older group was much more likely to be widowed and the younger group never married).

### Internet Use and Correlates

Table 2 shows participants’ Internet use patterns. Almost 60% of all participants had never used the Internet, 20% had used it before, and 20% were currently using it. Internet use did differ by age, with a higher rate of use for younger adults. For example, 38% of those under age 55 and 28% of those aged 55-59 were current users, while less than 15% of those 70 years and older were current users; however, only 10% of those 75-79 years old were current users while close to 15% of those 80-89 years were current users. As expected, those 90 years and older had the smallest proportion of current users (less than 9%). The proportion of previous users also varied by age but with higher rates of discontinued use among younger than older adults. A majority of previous users (76% of the under age 60 group and 60.8% of the 60 and up group) reported their inability to afford an Internet subscription and/or a new computer. Some reported that their old computers were no longer working/broken. In addition, 13.3% of the younger group and 14.2% of the older group reported they stopped using computers/the Internet because of disability, pain, or vision impairment. 9.3% of the younger group and 18.3% of the older group reported they no longer use the Internet because they do not need it. As well, 1.3% of the younger group and 1.7% of the older group reported discontinuing use because the Internet was not helpful. 5% of the older group reported they just moved and needed to reassemble/reconnect their computer before they could use it again, and one person (77 years old) reported that he did not have enough time for the Internet. Age group differences in reasons for discontinuing use were not significant (Pearson χ^2^_4_=7.91, *P*=.10)

Table 3 shows the results of multinomial logistic regression analysis. Among all participants, having never used the Internet, as opposed to current use, was significantly associated with older age, being black or Hispanic (as opposed to being nonHispanic white), and having lower income. Odds ratios show that blacks were 4.4 times and Hispanics were 4.7 times more likely than non-Hispanic whites to have never used the Internet (OR 4.41; 95% CI 2.82-6.91, *P*<.001 for blacks and OR 4.69; 95% CI 2.61-8.44, *P*<.001 for Hispanics) when other variables were held constant. The likelihood of having never used the Internet decreased by 36% for every one unit increase in income-to-needs ratio (OR 0.36; 95% CI 0.27-0.49, *P*<.001). Discontinued use, as opposed to current use, was not significantly associated with age; however, it was significantly associated with ethnicity, with blacks almost twice and Hispanics almost three times more likely to have discontinued Internet use (OR 1.79; 95% CI 1.08-2.95, *P*<.05 for blacks and OR 2.86; 95% CI 2.53-5.35, *P*<.001 for Hispanics). In addition, the likelihood of discontinued use decreased by 62% with every one unit increase in income-to-needs ratio (OR 0.62; 95% CI 0.45-0.86, *P*<.001).

Multinomial logistic regression results for older adults only show that the likelihood of having never used the Internet decreased by 62% with a self-reported depression diagnosis (OR 0.62; 95% CI 0.39-0.98, *P*<.05), suggesting that depressed older adults were more likely to have used the Internet than their peers without this diagnosis. The likelihood of discontinued Internet use decreased by 53% for older adults living alone (OR 0.53; 95% CI 0.31-0.92, *P*<.05), indicating that older adults living alone are more likely than those living with others to use the Internet.

**Table 1:** Sociodemographic and health and mental health characteristics by age group.

		All (N=980, 100%)	Under 60 (n=217, 22.1%)	60 and older (n=763, 77.9%)	*P* value^d^
**Age, mean (SD)**		71.31 (13.43)	53.83 (6.04)	76.57 (9.83)	<.001
**Gender (%)**					.40
	Male	29.5	31.8	28.8	
	Female	70.5	68.2	71.2	
**Race/ethnicity (%)**					.33
	Non-Hispanic white	41.7	39.6	42.3	
	Black/African American	36.2	38.2	35.6	
	Hispanic	20.8	19.8	21.1	
	Other	1.2	2.3	0.9	
**Primary language spoken at home (%)**					.24
	English	86.8	89.9	84.9	
	English/Spanish	11.0	8.9	11.7	
	Spanish				
	Other				
**Marital status (%)**					<.001
	Married	17.7	13.4	18.9	
	Widowed	33.9	6.9	41.5	
	Divorced/separated	34.7	48.4	30.8	
	Never married	13.8	31.1	8.8	
**Living arrangement (%)**					.02
	Live alone	58.0	56.7	58.3	
	Live with spouse	16.7	12.0	18.1	
	Live with other adult	21.2	24.9	20.2	
	Live with dependent child	4.1	6.5	3.4	
**Income-to-needs ratio, mean (SD)**		1.15 (0.65)	0.95 (0.51)	1.21 (0.67)	<.001
**No. of chronic medical conditions** ^a^ **, mean (SD)**		3.03 (1.54)	2.79 (1.62)	3.10 (1.50)	.01
**No. of ADL impairment** ^b^ **, mean (SD)**		1.69 (1.41)	1.68 (1.65)	1.69 (1.34)	.89
**No. of IADL impairment** ^c^ **, mean (SD)**		3.26 (1.49)	2.95 (1.58)	3.34 (1.45)	.001
**Diagnosis of depression (%)**		41.9	63.1	35.9	<.001
**Diagnosis of anxiety (%)**		23.1	37.8	18.9	<.001
**Diagnosis of severe mental illness (%)**		12.2	28.2	7.8	<.001

^a^Includes arthritis, hypertension, diabetes, heart disease, lung disease, kidney disease, stroke, and cancer.

^b^Includes moderate to severe impairment in feeding/eating, dressing, grooming, bathing, toileting, and transferring from bed to chair.

^c^Includes moderate to severe impairment in cleaning, doing laundry, preparing meals, shopping, taking medication, and making telephone calls.

^d^ *P* denotes difference between the two age groups.

**Table 2:** Internet use status by age group (%).

Age group		Distribution, n (%)	Never user	Previous user	Current user
All ages		980 (100)	59.6	19.9	20.5
Under 60^a^		217 (22.1)	31.8	34.6	33.6
60 and older		763 (77.9)	67.5	15.7	16.8
**Under 55** ^b^		118 (12.0)	27.1	34.7	38.1
	55-59	99 (10.1)	37.4	34.3	28.3
	60-64	117 (11.9)	50.4	23.1	26.5
	65-69	118 (12.0)	48.3	27.1	24.6
	70-74	111 (11.3)	67.6	18.0	14.4
	75-79	118 (12.0)	70.3	19.5	10.2
	80-84	121 (12.3)	76.9	8.3	14.9
	85-89	110 (11.2)	80.0	5.5	14.5
90 and older		68 (6.9)	88.2	2.9	8.8

^a^Age group difference in Internet use patterns was significant: Pearson χ^2^_2_=98.68, *P*<.001.

^b^Age group difference in Internet use patterns was significant: Pearson χ^2^_14_=153.53, *P*<.001.

### Internet Use Patterns and Activities Among Current Users

Table 4 shows that 86% of the younger group and 95% of the older group had an Internet connection at home, while the rest used an Internet connection available at their apartment complex, family/friends’ home, and other places including a store with wireless services. The age groups differed significantly in terms of their Internet connection sites (*P*=.02), with the older group more likely to connect at home; however, they did not differ in frequency of Internet use. A little more than half of both younger and older groups used the Internet daily, a little over 20% used it every few days, 10-11% used it once a week, and the rest used it less often than weekly. For both groups, sending and receiving email was the most popular Internet activity, followed by research on nonhealth- and health-related information. A little more than 75% of the younger group reported health-related information searches, but only 55% of the older group reported the same (*P*=.01). Significant age group differences were also found in Internet use for playing games (56% of the younger group vs 39% of the older group, *P*=.03), watching videos (49% vs 27%, *P*=.01), and for social network or dating sites (48% vs 20%, *P*<.001). Although not statistically significant, almost half of the younger group engaged in online goods purchases and banking/bill paying and reading online papers/news, magazines and books, while 35-39% of the older group did the same. “Other” Internet use (not reported in Table 4) included collecting coupons, looking at used car/motorcycle pictures on craigslist, looking at other photos, video chats using “Skype and Tango”, checking lottery winning numbers; using it as Yellow pages/directory, listening to the radio, and visiting religious websites. Both age groups reported that they had a relatively easy time finding the information they were looking for on the Internet: mean 2.14 (SD 1.08) for the younger group and mean 2.27 (SD 1.15) for the older group, *t*=0.82, *P*=.41), suggesting that they felt confident about their search skills. However, many reported discomfort in using the computer/Internet due to physical, functional, and vision-related limitations. The problems they reported included arthritic pain in the fingers, neck and back pain, neuropathy, difficulty typing due to other disability, chronic fatigue and other medical conditions that interfere with their ability to concentrate and sit for a long period of time, and glaucoma and other vision problems. In addition, a few older adults said they were “too old to learn new things,” were fearful of radiation exposure from the computer, and had insufficient reading comprehension to effectively use Internet resources.

**Table 3:** Correlates of Internet use and nonuse: odds ratios (OR) from multinomial regression results.

	All ages (n=968)		Older adults only (n=756)	
	Never use vs current use OR (95% CI)	Previous use vs current use OR (95% CI)	Never use vs current use OR (95% CI)	Previous use vs current use OR (95% CI)
Age	1.09^a^ (1.07-1.11)	1.01 (0.99-1.03)	1.08^a^ (1.06-1.11)	0.99 (0.96-1.02)
Male	1.00	1.00	1.00	1.00
Female	1.05 (0.70-1.57)	1.16 (0.74-1.82)	0.94 (0.58-1.53)	1.31 (0.73-2.36)
Non-Hispanic white	1.00	1.00	1.00	1.00
Black	4.4^a^ (2.82-6.91)	1.79^c^ (1.08-2.95)	5.13^a^ (2.96-8.92)	2.12^c^ (1.09-4.14)
Hispanic	4.69^a^ (2.61-8.44)	2.86^a^ (2.53-5.35)	7.63^a^ (3.28-17.74)	5.62^a^ (2.25-14.06)
English-speaking	1.00	1.00	1.00	1.00
Spanish-speaking	0.77 (0.14-4.16)	0.66 (0.10-4.44)	0.51 (0.06-4.49)	0.38 (0.03-4.97)
Not living alone	1.00	1.00	1.00	1.00
Living alone	0.82 (0.56-1.21)	0.74 (0.48-1.13)	0.78 (0.48-1.24)	0.53^c^ (0.31-0.92)
Income-to-needs ratio	0.36^a^ (0.27-0.49)	0.62^b^ (0.45-0.86)	0.34^a^ (0.24-0.48)	0.65 (0.45-0.94)
No depression diagnosis	1.00	1.00	1.00	1.00
Depression diagnosis	0.74 (0.50-1.10)	0.97 (0.63-1.52)	0.62^c^ (0.39-0.98)	0.64 (0.37-1.12)
No anxiety diagnosis	1.00	1.00	1.00	1.00
Anxiety diagnosis	0.94 (0.60-1.49)	1.34 (0.83-2.16)	1.11 (0.63-1.96)	1.53 (0.80-2.92)
No. of medical conditions	0.93 (0.83-1.05)	0.88^d^ (0.77-1.01)	0.92 (0.79-1.07)	0.83^c^ (0.70-0.99)
No. of ADL/IADL impairment	1.07^d^ (1.00-1.15)	1.04 (0.96-1.12)	1.08 (0.98-1.17)	1.06 (0.96-1.18)
				
-2 LL Model χ^2^_22_	307.90		219.02	
*P*	<.001		<.001	
Pseudo *R* ^2^ (Cox and Snell; Nigelkerke)	0.27; 0.32		0.25; 0.31	

^a^ *P*<.001.

^b^ *P*<.01.

^c^ *P*<.05.

^d^ *P*<.06.

**Table 4:** Internet use patterns and activities among current users.

		Under 60 n=217 (22.1%)	60 and older n=763 (77.9%)	*P* value^b^
**Location of Internet connection**				.02
	At home	86.3	95.2	
	Apartment complex	4.1	4.0	
	Family/friend’s house	2.7	0.8	
	Other (eg, store with Wi-Fi)	6.8	0	
**Frequency of use**				.97
	Daily	54.8	53.2	
	Every few days	23.3	21.0	
	Once a week	9.6	11.3	
	A few times a month	6.8	7.3	
	Once a month of less often	5.5	7.3	
**Type of use**				
	Research health-related information	75.3	54.7	.01
	Research information about other topics	76.7	66.4	.15
	Send/Receive email	78.1	76.6	.86
	Buy product online	49.3	35.2	.05
	Do banking /paying bills online	46.6	39.1	.30
	Read papers/news, magazines, and books	50.7	36.7	.07
	Play games	56.2	39.1	.03
	Watch videos (eg, YouTube)	49.3	27.3	.01
	Use social network or dating site (eg, Facebook, Match.com)	47.9	19.5	<.001
Self-reported ease of Internet search^a^, mean (SD)		2.14 (1.08)	2.27 (1.15)	.41
**Problems causing difficulty using the computer/Internet**				
	Pain in the limbs, neck, and back	60.3	39.8	.01
	Unsteady hands	49.3	42.2	.38
	Difficulty concentrating (due to chronic fatigue; recent stroke)	45.2	39.8	.46
	Difficulty sitting for a long period of time	50.7	46.1	.30
	Vision problems (tired eyes, poor vision…)	52.1	46.1	.46
	Other (“too old to learn new things”, fear of radiation exposure, amputated hands, hands not usable, comprehension difficulty)	2.7	4.9	.28

^a^On a scale of 1-5, with lower scores suggesting higher level of ease and lower-level of difficulty.

^b^ *P* denotes difference between the two age groups.

### eHealth Literacy, Attitudes Toward Computer/Internet, and Correlates

Table 5 shows eHEALS scores for current users and ATC/IQ scores for never users by age group. eHEALS scores suggest that self-rated eHealth literacy for both age groups, on average, are at a neutral (ie, “undecided”) level, although the younger group’s score was significantly higher than the older group’s: mean 3.53 (SD 0.76) vs mean 3.22 (SD 0.85), *t*=2.57, *P*=.01. Both age groups scored slightly higher on the Internet’s usefulness in helping them make decisions about their health (mean 3.67 (SD 1.11) and mean 3.41 (SD 1.28), *t*=1.48, *P*=.14) and about the importance of being able to access health resources on the Internet (mean 3.75 (SD 1.16) and mean 3.43 (SD 1.32), *t*=1.74, *P*=.08). eHEALS scores and supplemental item scores were highly correlated (*r*=0.67, *P*<.001 for eHEALS and perceived usefulness and *r*=0.69, *P*<.001 for eHEALS and perceived importance). Perceived usefulness scores and perceived importance scores were also highly correlated (*r*=0.79, *P*<.001). However, both groups expressed a lower level of certainty/comfort about joining online health discussion groups and exchanging emails with other participants, with less willingness among older adults: mean 2.89 (SD 1.23) for the younger group and mean 2.52 (SD 1.30) for the older group, *t*=2.01, *P*=.05.

Both ATC/IQ efficacy and interest scores, on average, suggest a neutral (undecided) level of efficacy and interest. The younger group’s efficacy score was slightly and significantly higher than the older group’s: mean 3.34 (SD 0.77) vs 3.01 (SD 0.98), *t*=2.66, *P*=.01. However, there was no age group difference in interest level: mean 3.33 (SD 0.88) vs 3.23 (SD 0.85), *t*=0.88, *P*=.38. Willingness to use online health information (if someone taught them how to use the computer/Internet) was also at a neutral level, with slightly and significantly higher scores for the younger than older group: mean 3.52 (SD 1.05) vs 3.03 (SD 1.22), *t*=3.18, *P*=.01. Comfort with joining online health discussion groups and exchanging emails with other participants was also slightly and significantly higher for younger than older adults: mean 2.81 (SD 1.20) vs 2.44 (SD 1.17), *t*=2.50, *P*=.01.

Another way to examine eHEALS and ATC/IQ scores is by the proportion of those who agreed (rating of 4) or strongly agreed (rating of 5) with the scale items. Among respondents, 35.7% of the younger group and 29.0% of the older group had an average score of 4+ on the 8-item eHEALS, 65.7% of the younger and 63.3% of the older groups rated perceived usefulness as 4 or 5, 68.5% of the younger and 62.5% of the older groups rated perceived importance 4 or 5, and 39.7% and 31.3% of the respective age groups rated their comfort level with joining online health discussion groups and exchanging emails with other participants as 4 or 5. Further analysis also showed that 40.5% of the younger group and 27.3% of the older group scored 4+ on the ATC/IQ efficacy subscale, 36.2% of the younger group and 28.8% of the older group scored 4+ on the ATC/IQ interest subscale, 68.1% and 48.4% of the respective age groups scored 4 or 5 on their willingness to try accessing online health information, and 40.5% and 25.0% of the respective age groups scored their comfort level with joining online health discussion groups and exchanging emails with other participants as 4 or 5.

As expected, Table 6 shows that eHEALS scores among current Internet users of all ages were inversely associated with age and computer/Internet use frequency, with these variables alone explaining 27% of the variance in eHEALS scores. No other sociodemographic and health/mental health characteristics were associated with eHEALS scores. Among the older age group, however, depression was also inversely associated with eHEALS scores: B=-0.33 (SE 0.14), *P*=.02. The model adjusted *R* ^2^ was .36.

As Table 7 shows, ATC/IQ efficacy among never users of all ages was positively associated with living alone, income-to-needs ratio, and the number of medical conditions and inversely associated with age, Hispanic ethnicity, and Spanish as the primary language spoken at home. Among the older age group, being black was also marginally positively associated with higher efficacy scores: B=0.19 (SE 0.10), *P*=.057. ATC/IQ interest among never users of all ages was positively associated with being black but inversely associated with age, Hispanic ethnicity (marginally), and Spanish as the primary language spoken at home. Among the older age group, Hispanic ethnicity and Spanish as the primary language were not significant factors, while living alone was marginally positively associated with higher interest scores: B=0.14 (SE 0.07), *P*=.066. Given the low *R* ^2^ statistics (15% for efficacy and 8-10% for interest), it appears that variables not captured by the participants’ sociodemographic and health/mental health characteristics may influence ATC/IQ efficacy and interest.

**Table 5:** eHealth Literacy (eHEALS), Attitudes Toward Computer/Internet (ATC/IQ), and willingness to use health information searches and online health discussion groups: mean and standard deviation of the mean.

	Current User		Never User	
	Under 60 (n=73)	60 and older (n=218)	Under 60 (n=69)	60 and older (n=515)
8-item eHEALS	3.53 (0.76)^b^	3.22 (0.85)^b^		
Perceived usefulness	3.67(1.11)	3.41 (1.28)		
Perceived importance	3.75 (1.16)	3.43 (1.32)		
ATC/IQ efficacy			3.34 (0.77)^a^	3.01 (0.98)^a^
ATC/IQ interest			3.33 (0.88)	3.23 (0.85)
Willingness to try online health information (if someone can teach me how)			3.52 (1.05)^a^	3.03 (1.22)^a^
Comfort with online health discussions groups and email exchanges with other participants	2.89 (1.23)	2.52 (1.30)	2.81 (1.20)^b^	2.44 (1.17)^b^

^a^ *P*<.01.

^b^ *P*<.05; denotes difference between the two age groups.

**Table 6:** Correlates of eHEALS among current Internet users.

	All ages (n=198) B (SE)	Older adults only (n=128) B (SE)
Age	-0.02 (0.01)^a^	-0.03 (0.01)^a^
Female	0.14 (0.11)	-0.01 (0.14)
Black	0.17 (0.14)	0.13 (0.18)
Hispanic	-0.27 (0.19)	-0.42 (0.29)
Spanish-speaking	-0.03 (0.76)	0.02 (0.78)
Live alone	0.09 (0.11)	0.18 (0.14)
Income-to-needs ratio	0.11 (0.07)	0.11 (0.08)
Diagnosis of depression	-0.17 (0.11)	-0.33 (0.14)^c^
Diagnosis of anxiety	0.11 (0.13)	0.03 (0.17)
No. of medical conditions	0.00 (0.03)	0.01 (0.04)
No. of ADL/IADL impairment	-0.01 (0.02)	0.02 (0.03)
Use Internet a few times a week	-0.44 (0.14)^b^	-0.36 (0.18)^c^
Use Internet once a week or less often	-0.88 (0.13)^a^	-1.01 (0.16)^a^
*R* ^2^	0.32	0.40
Adjusted *R* ^2^	0.27	0.36
SE	0.71	0.70
df (*P*)	13 (<.001)	13 (<.001)

^a^ *P*<.001.

^b^ *P*<.01.

^c^ *P*<.05.

**Table 7:** Correlates of ATC/IQ efficacy and interest among those who never used the Internet.

	ATC/IQ efficacy		ATC/IQ interest	
	All ages (n=577) B (SE)	Older adults only (n=509) B (SE)	All ages (n=577) B (SE)	Older adults only (n=509) B (SE)
Age	-0.02 (0.00)^a^	-0.02 (0.01)^a^	-0.01 (0.00)^b^	-0.02 (0.00)^a^
Female	0.09 (0.08)	0.13 (0.09)	0.11 (0.08)	0.12 (0.08)
Black	0.14 (0.09)	0.19 (0.10)^d^	0.24 (0.08)^b^	0.32 (0.09)^a^
Hispanic	-0.40 (0.11)^a^	-0.34 (0.12)^b^	-0.17 (0.10)^d^	-0.12 (0.10)
Spanish-speaking	-0.68 (0.20)^a^	-0.65 (0.21)^b^	-0.40 (0.19)^c^	-0.31 (0.19)
Living alone	0.15 (0.08)^c^	0.21 (0.08)^c^	0.06 (0.06)	0.14 (0.07)^d^
Income-to-needs ratio	0.15 (0.07)^c^	0.21 (0.07)^b^	0.05 (0.08)	0.11 (0.07)
Diagnosis of depression	-0.07 (0.08)	-0.06 (0.09)	-0.05 (0.08)	-0.04 (0.08)
Diagnosis of anxiety	0.04 (0.10)	0.00 (0.11)	-0.04 (0.09)	-0.03 (0.10)
No. of medical conditions	0.05 (0.03)	0.03 (0.03)	0.03 (0.02)	0.01 (0.03)
No. of ADL/IADL impairment	-0.00 (0.02)	-0.01 (0.02)	-0.01 (0.01)	-0.01 (0.02)
*R* ^2^	0.17	0.17	0.10	0.12
Adjusted *R* ^2^	0.15	0.15	0.08	0.10
SE	0.87	0.90	0.82	0.81
df (*P*)	11 (<.001)	11 (<.001)	11 (<.001)	11 (<.001)

^a^ *P*<.001.

^b^ *P*<.01.

^c^ *P*<.05.

^d^ *P*<.07.

## Discussion

Many previous studies describe the digital divide between older and younger people, with the divide being greater for those who are racial/ethnic minorities and of lower socioeconomic status (SES) [19,22,25,28,34]. Much research has also been done regarding the psychological, functional, and educational barriers that prevent many older adults from joining the digital age and taking advantage of the many benefits that Internet technology can offer [18,20,35,36]. Most previous research included older adults with varying degrees of SES and functional abilities, and few focused on those with low-incomes and disabilities. This study examined Internet use patterns, eHealth literacy, and attitudes toward computer/Internet among a large sample of low-income homebound older adults and compared them to a younger group of low-income homebound individuals. Because of their disabilities, none worked for pay and all received home-delivered meals; thus, any computer/Internet use in which they engaged would have been exclusively for their personal use.

As expected, this vulnerable group of individuals had low rates of Internet use—34% of the younger group (under 60 years) and 17% of the older group (60 years and older). For the most part, age was inversely associated with Internet use, with lower use rates among those with more advanced age. However, this study also found that the numbers who had to discontinue Internet use (35% of the younger group and 16% of the older group) were as many as those who were currently using the Internet. The primary reasons for discontinuation were the cost of an Internet connection and/or replacing a nonfunctional computer and disability, pain, and vision problems. For those who could not afford an Internet subscription, computer, or other necessary equipment, it appears that they could have continued using the Internet if affordability was not a barrier.

The study also found that even within this group of low-income homebound persons, racial/ethnic minorities and those with lower income were much less likely to use the Internet, indicating the persistent negative effects of racial/ethnic minority status and low SES on digital inclusion. Multivariate analyses also identified the number of chronic medical conditions and ADL/IADL impairments as marginally significant correlates of Internet use. As expected, ADL/IADL impairments were barriers to Internet use; however, higher numbers of chronic medical conditions were positively associated with current Internet use as opposed to discontinued use. While having chronic medical conditions seems to promote Internet use, it is not clear if this results from the need to obtain medical information or manage ones’ health care or whether having more chronic conditions results in greater isolation, making the Internet important in maintaining social connections. Also important was that a self-reported diagnosis of depression or anxiety was not a significant correlate, suggesting that these mental health conditions did not inhibit Internet use. Thus, H1 was partially supported.

Regardless of age, about three-fourths of Internet users went online either daily or every few days, suggesting that a majority of these low-income, homebound Internet users integrated Internet technology in their usual routines. In this respect, they were not different from the general US population. The aforementioned national survey done in 2012 found that 70% of US adults aged 65 and older used the Internet on a typical day and 67% of all American adults aged 18 and older did so [19]. Younger persons in our sample reported utilizing the Internet for a wider variety of activities, taking advantage of the many benefits the Internet offers. Older users were not taking full advantage of the Internet’s multiple benefits, as only about 55% did research on health-related information and only one-fifth were using the Internet for social networking purposes. This may reflect their reports that searching and finding the information they needed fell somewhere between “sometimes easy” and “not so easy.” Although most users in the older age group used the Internet daily or almost daily, they appear to have somewhat low levels of confidence in their Internet search skills, which was reflected in their average eHEALS score being significantly lower than in the younger age group. In multivariate analyses, eHEALS scores were negatively associated with age, meaning those of older ages had lower perceptions of their eHealth self-efficacy. Better news is that self-efficacy was significantly determined by the frequency of Internet use, a finding consistent with previous studies that training and practicing Internet skills increases older adults’ computer-related self-efficacy [18,29-32]. As hypothesized, eHEALS scores in the older age group were also inversely associated with a depression diagnosis, which may reflect the generally negative self-evaluation among depressed older adults, even though older adults with depression were more likely to use the Internet than those without this diagnosis. These findings support H2. The low-level of eHealth efficacy among both age groups may also have been responsible for respondents’ lack of willingness to join online health discussion groups, although the lack of willingness may also have stemmed from privacy concerns related to the exchange of emails with other participants or warnings to avoid being taken advantage of online. Some low-income homebound older adults may have faced discrimination or mistreatment by others throughout their lives, and as a result, they may have been cautious about connecting with strangers online, although they perceived the usefulness and importance of the Internet-based health resources.

Those who never used the Internet did not express aversion to learning to use it and believed they could do so, although older respondents were slightly less confident than their younger counterparts. In addition to age, Hispanics and those who primarily spoke Spanish at home expressed lower levels of computer/Internet efficacy, though it is not clear why Hispanics, even controlling for Spanish speaking, had a lower level of efficacy. Participants who lived alone and participants, including those in the older age group, with higher incomes had higher efficacy levels. Those living alone may have more confidence in general given their ability to live independently and may also feel a greater need to use email and other Internet technology that can connect them to others.

Although bivariate analyses showed that low-income homebound older adults’ computer/Internet interest did not differ significantly from their younger counterparts, multivariate analyses again showed that older age was a significant negative factor. Although blacks were less likely to use the Internet than nonHispanic whites, black older adults and older adults who lived alone expressed greater interest in computer/Internet use than their nonHispanic white counterparts and those who lived with someone else. Hispanics were also less likely than nonHispanic whites to use the Internet, but ethnicity and Spanish-language use were not significant factors for older adults’ level of interest. Thus, H3 was partially supported.

A few study limitations should be noted. First, despite the large sample size, the survey participants were selected from a geographically limited area, which may limit the findings’ generalizability. Second, although case managers confirmed participants’ reported health and mental health conditions using medication lists, these diagnoses by themselves may not be equated with symptom manifestations and severity, especially in the case of depression, anxiety, and severe mental illness. Pharmacotherapy may have effectively reduced symptoms for some people. Thus, actual assessment of current symptoms would have allowed more accurate evaluation of their influence. Third, a potentially important omission in the agency assessment dataset was the clients’ level of education, which could have provided a more comprehensive picture of participants’ sociodemographic characteristics and their level of disadvantage.

Despite its limitations, the present study is the first to provide a detailed description of Internet use and nonuse among low-income, disabled, and homebound older and younger adults. We found very low rates of Internet use compared to the US population, either due to lack of exposure to computer and Internet technology, lack of financial resources to obtain computers and access technology for personal use, or medical conditions, disabilities, and associated pain that restrict use. However, the findings also provide hope for reducing the digital divide because while blacks and Hispanics were less likely to be current Internet users, black older adults expressed greater interest and Spanish-speaking adults were no less interested than nonHispanic whites in computer/Internet use. Internet technology can offer multiple benefits to vulnerable homebound individuals and may contribute to reducing health disparities among other disabled homebound individuals. Thus, improving access to Internet technology that can enhance health and well-being should be viewed as a social justice issue. As health care systems increasingly rely on Internet technology to manage patients’ records, communicate with them, and provide care, it will be necessary for patients who want greater involvement in their health care to become proficient in using health information technology. For example, access to one’s electronic health records and being able to communicate more readily with health care providers may go a long way in improving patient compliance with treatment regimens, engaging patients in their treatment, and increasing their control over what is happening to them. Those, such as homebound individuals who often have multiple health needs, can benefit the most from becoming well versed at using these tools.

Until the digital divide eventually disappears due to younger Americans’ nearly universal exposure to computer use and Internet technology, steps can be taken to increase Internet use among today’s older adults, including those who are disadvantaged, through social policy, technology/equipment design, and training/education. First, in the social policy domain, offering low-income persons technology subsidies/allowances may help them join the digital age, and government agencies and nongovernmental organizations (NGOs) could recycle and refurbish the many computers sitting idle or disposed of each year for older adults’ use. Such government-provided allowances and NGO efforts may result in cost effectiveness if they enable people to live independently, reduce their dependence on informal and formal support, and increase their quality of life. Second, to encourage Internet use among individuals with substantial disabilities, technology should be designed to be as user-friendly as possible [41]. For example, touch screens tend to be more usable than keyboards for older adults with arthritic pains in their fingers/hands. However, future technology designs should go far beyond many current innovations and provide personalized technology systems to facilitate use even among those with a high degree of disability, such as greater access to voice systems for people with low literacy levels or who are visually impaired. Third, older adults who remain reluctant to use the Internet due to low technology-related self-efficacy may be motivated to embrace its use through demonstrations and education. The present study suggests that exposure and practice (ie, frequent use) increases Internet skill efficacy regardless of income level and disability. Many younger people’s computer and Internet skills could be used by employing them through volunteer work or paid employment to teach older adults how to use email, surf the Web, and engage in social networking and health-related tasks. Especially for low-income homebound older adults who have not been exposed to computer/Internet technology but whose needs are substantial, the multiple benefits of computer/Internet use need to be emphasized and equipment and training provided to facilitate their use.

Future studies should test the extent to which providing computer equipment (especially devices that are easiest to use), Internet connections, computer applications, and training to use them increase interest, use, and efficacy and are associated with improved health, mental health, and other well-being outcomes. Studies should also test the types of devices and applications that homebound adults may be most interested in using and the features that facilitate use and better outcomes for this population.

## Abbreviations

ADL/IADL: activities of daily living and instrumental activities of daily living
ATC/IQ: Attitudes Toward Computer/Internet Questionnaire
eHEALS: eHealth Literacy Scale
HDM: home-delivered meals
NGOs: nongovernmental organizations
SES: socioeconomic status

## References

[1] United States Census Bureau (2009). Statistical Table Based on data from the 2009 American Community Survey.

[2] Centers for Medicare and Medicaid Services (2012). Table 7.2, Persons Served, Visits, Total Charges, Visit Charges, and Program Payments for Medicare Home Health Agency Services: Calendar Year 2011.

[3] Barrett A, Schimmel J (2012). Issue Brief No 2.

[4] Altchuler N, Schimmel J (2010). Issue Brief No. 1.

[5] Colello KJ (2011). Congressional Research Service Report for Congress.

[6] Choi NG, Teeters M, Perez L, Farar B, Thompson D (2010). Severity and correlates of depressive symptoms among recipients of meals on wheels: age, gender, and racial/ethnic difference. Aging Ment Health.

[7] Ell K, Unützer J, Aranda M, Sanchez K, Lee PJ (2005). Routine PHQ-9 depression screening in home health care: depression, prevalence, clinical and treatment characteristics and screening implementation. Home Health Care Serv Q.

[8] Sirey JA, Bruce ML, Carpenter M, Booker D, Reid MC, Newell KA, Alexopoulos GS (2008). Depressive symptoms and suicidal ideation among older adults receiving home delivered meals. Int J Geriatr Psychiatry.

[9] Grady B, Myers KM, Nelson EL, Belz N, Bennett L, Carnahan L, Decker VB, Holden D, Perry G, Rosenthal L, Rowe N, Spaulding R, Turvey CL, White R, Voyles D, American Telemedicine Association Telemental Health Standards and Guidelines Working Group (2011). Evidence-based practice for telemental health. Telemed J E Health.

[10] van den Berg N, Schumann M, Kraft K, Hoffmann W (2012). Telemedicine and telecare for older patients--a systematic review. Maturitas.

[11] Wilcox ME, Adhikari NK (2012). The effect of telemedicine in critically ill patients: systematic review and meta-analysis. Crit Care.

[12] (2009). Internet use among midlife and older adults: an AARP bulletin Poll.

[13] Crabb RM, Rafie S, Weingardt KR (2012). Health-related internet use in older primary care patients. Gerontology.

[14] Czaja SJ, Lee CC (2006). The impact of aging on access to technology. Univ Access Inf Soc.

[15] Gatto SL, Tak SH (2008). Computer, Internet, and E-mail Use Among Older Adults: Benefits and Barriers. Educational Gerontology.

[16] Russell C, Campbell A, Hughes I (2008). Ageing, social capital and the Internet: findings from an exploratory study of Australian 'silver surfers'. Australas J Ageing.

[17] Sum S, Mathews RM, Hughes I (2009). Participation of older adults in cyberspace: how Australian older adults use the Internet. Australas J Ageing.

[18] Xie B (2011). Effects of an eHealth literacy intervention for older adults. J Med Internet Res.

[19] Zickuhr K, Madden M Pew Research Center's Internet & American Life Project.

[20] Adams N, Stubbs D, Woods V (2005). Psychological barriers to Internet usage among older adults in the UK. Med Inform Internet Med.

[21] Carpenter BD, Buday S (2007). Computer use among older adults in a naturally occurring retirement community. Computers in Human Behavior.

[22] Choi N (2011). Relationship between health service use and health information technology use among older adults: analysis of the US National Health Interview Survey. J Med Internet Res.

[23] Dreisbach N, Axler F, Kotranski L, Weingartner RM, Ingerman S, Ramirez N, Klein G (2011). Assessing the digital divide among older adults residing in an urban and suburban metropolitan area. Proceedings of the 139th Annual Meeting of the American Public Health Association.

[24] Slegers K, van Boxtel MPJ, Jolles J (2012). Computer use in older adults: determinants and the relationship with cognitive change over a 6 year episode. Comput Hum Behav.

[25] Werner JM, Carlson M, Jordan-Marsh M, Clark F (2011). Predictors of computer use in community-dwelling, ethnically diverse older adults. Hum Factors.

[26] Charness N, Boot WR (2009). Aging and information technology use. Curr Dir Psychol Sci.

[27] Choi NG, DiNitto DM (2013). Different types of Internet use among older adults:. Association with health needs, psychological capital, and social capital.

[28] Berry R (2011). Older people and the Internet.

[29] Chu A, Huber J, Mastel-Smith B, Cesario S (2009). "Partnering with Seniors for Better Health": computer use and Internet health information retrieval among older adults in a low socioeconomic community. J Med Libr Assoc.

[30] Czaja SJ, Sharit J (1998). Age differences in attitudes toward computers. J Gerontol B Psychol Sci Soc Sci.

[31] Wild KV, Mattek NC, Maxwell SA, Dodge HH, Jimison HB, Kaye JA (2012). Computer-related self-efficacy and anxiety in older adults with and without mild cognitive impairment. Alzheimers Dement.

[32] Lagana L (2008). Enhancing the Attitudes and Self-Efficacy of Older Adults Toward Computers and the Internet: Results of a Pilot Study. Educ Gerontol.

[33] Norman CD, Skinner HA (2006). eHEALS: The eHealth Literacy Scale. J Med Internet Res.

[34] Czaja SJ, Sharit J, Lee CC, Nair SN, Hernández MA, Arana N, Fu SH (2013). Factors influencing use of an e-health website in a community sample of older adults. J Am Med Inform Assoc.

[35] Jensen JD, King AJ, Davis LA, Guntzviller LM (2010). Utilization of internet technology by low-income adults: the role of health literacy, health numeracy, and computer assistance. J Aging Health.

[36] Neter E, Brainin E (2012). eHealth literacy: extending the digital divide to the realm of health information. J Med Internet Res.

[37] Xie B (2011). Older adults, e-health literacy, and collaborative learning: An experimental study. J Am Soc Inf Sci.

[38] Bear GG, Richards HC, Lancaster P (1995). Attitudes Toward Computers: Validation of a Computer Attitudes Scale. Journal of Educational Computing Research.

[39] Jay GM, Willis SL (1992). Influence of direct computer experience on older adults' attitudes toward computers. J Gerontol.

[40] Xie B, Bugg JM (2009). Public library computer training for older adults to access high-quality Internet health information. Libr Inf Sci Res.

[41] Wagner N, Hassanein K, Head M (2010). Computer use by older adults: A multi-disciplinary review. Comput Hum Behav.

